# PCR detection of human herpesviruses in colonic mucosa of individuals with inflammatory bowel disease: Comparison with individuals with immunocompetency and HIV infection

**DOI:** 10.1371/journal.pone.0184699

**Published:** 2017-09-13

**Authors:** Takayuki Shimada, Naoyoshi Nagata, Koki Okahara, Akane Joya, Tsunefusa Hayashida, Shinichi Oka, Toshiyuki Sakurai, Junichi Akiyama, Naomi Uemura, Hiroyuki Gatanaga

**Affiliations:** 1 Department of Gastroenterology and Hepatology, National Center for Global Health and Medicine, Tokyo, Japan; 2 AIDS Clinical Center, National Center for Global Health and Medicine, Tokyo, Japan; 3 Department of Gastroenterology and Hepatology, National Center for Global Health and Medicine, Kohnodai Hospital, Chiba, Japan; National Institute of Child Health and Human Development, UNITED STATES

## Abstract

**Background:**

Detection of human herpesviruses (HHVs) other than cytomegalovirus (CMV) in colonic mucosa of individuals with inflammatory bowel disease (IBD) remains unknown. This study identified eight HHVs in the colonic mucosa of individuals with IBD and compared the results with immunocompetent and human immunodeficiency virus (HIV)-infected individuals.

**Methods:**

A total of 89 individuals who had colorectal ulcer on colonoscopy were enrolled: 26 with immunocompetency (n = 26), 41 with IBD, and 22 with HIV infection. We examined the colonic ulcers for the presence of eight HHVs—herpes simplex virus (HSV)-1/2, varicella zoster virus (VZV), CMV, Epstein–Barr virus (EBV), HHV-6, HHV-7, and HHV-8—using mucosal PCR.

**Results:**

The IBD group had positivity rates of 0%, 0%, 0%, 53.7%, 24.4%, 39%, 39%, and 0% for HSV-1, HSV-2, VZV, EBV, CMV, HHV-6, HHV-7, and HHV-8, respectively. The positivity rates of EBV and CMV in colonic mucosa increased significantly in the order of the immunocompetent, IBD, and HIV groups (EBV: 23.1%, 53.7%, 72.7%, P for trend = 0.0005; CMV, 7.7%, 24.4%, 54.5%, P for trend = 0.0003, respectively), but no increase was found in the other HHVs. Median mucosal EBV DNA values in the immunocompetent, IBD, and HIV groups were 0, 76, and 287 copies/μg DNA, respectively (P for trend = 0.002). Corresponding median mucosal CMV DNA values were 0, 0, and 17 copies/μg DNA (P for trend = 0.0001). There was no significant difference in the positivity rates of the eight HHVs between ulcerative colitis and Crohn’s disease.

**Conclusion:**

The HHVs of EBV, CMV, HHV-6, and HHV-7, but not of HSV-1, HSV-2, VZV, or HHV-8, were identified in the colonic mucosa of IBD individuals. EBV and CMV in colonic mucosa was correlated with host immune status in increasing order of immunocompetent, IBD, and HIV-infected individuals.

## Introduction

Human herpesviruses (HHVs) are DNA viruses, and eight distinct members of the HHV family have been identified.[1] Most HHVs remain latent for a long time but can be reactivated and cause infection when host immunity is compromised.[2] Some studies have reported that reactivation of HHVs occurs in the colonic mucosa.[3]

The etiopathogenesis of inflammatory bowel disease (IBD) remain elusive, and accumulating evidence indicates that both mucosal immune dysregulation and genetics are involved in the disease process.[4] Individuals with IBD are often placed on long-term immunosuppressant drugs and may develop iatrogenic immunosuppression with consequent reactivation of HHVs in colonic mucosa. Cytomegalovirus (CMV) is frequently detected in the colonic mucosa of IBD individuals,[5] but detection of HHVs other than CMV in their colonic mucosa has not been reported.

A previous study showed that the detection rates of Epstein–Barr virus (EBV) and CMV were higher in IBD individuals than in immunocompetent (IC) individuals.[6] Another study showed that the detection rates of herpes simplex virus (HSV)-1 and HHV-6 were higher in stool samples of HIV-infected individuals than in those of IC individuals.[7] However, no previous studies have compared the detection rate of eight HHVs—HSV-1/2, varicella zoster virus (VZV), CMV, EBV, HHV-6, HHV-7, and HHV-8—among these three groups. For the identification and diagnosis of CMV in colonic mucosa, the European Crohn’s & Colitis Organization (ECCO) guidelines recommend mucosal PCR assay as a highly specific and sensitive method for verifying CMV infection in biopsy tissue.[8] Accordingly, we evaluated colonic mucosa by performing mucosal PCR assay for the eight HHVs.

This study aimed to determine the detection rates of the eight HHVs in individuals with IBD and examine the correlation between HHV infection rate and host immune status in IC, IBD, and HIV-infected individuals, as well as identify the difference in HHV infection rates between ulcerative colitis (UC) and Crohn’s disease (CD).

## Materials and methods

### Study design, setting, and participants

This retrospective observational study was conducted between 2011 and 2015 at the endoscopy unit of the National Center for Global Health and Medicine (NCGM), Tokyo, Japan. During the study period, we reviewed the data of 121 biopsy samples from 121 individuals who had colorectal ulcer confirmed on colonoscopy. The indications for colonoscopy were examination for lower gastrointestinal symptoms such as diarrhea, hematochezia, or abdominal symptoms. In clinical practice, we obtained pure biopsy samples from UC mucosa to differentiate between pathogenic bacteria-induced and virus-induced colorectal ulcer. To diagnose virus-induced ulcer in particular, we sought to definitively clarify the HHV detection rate in active inflammatory mucosa of the ulcer. All colorectal ulcers were diagnosed by high-resolution colonoscopy (Olympus AI260 Series, Olympus, Japan) with biopsy forceps (Boston Scientific single-use Radial JAW^TM^4 (2.8 mm) Boston, MA). We divided the study participants into IC, IBD, and HIV groups, respectively. The IC group comprised individuals with colonic ulcers due to non-virus infectious colitis, ischemic colitis, or nonspecific colitis, excluding those with HIV, IBD, renal insufficiency/dialysis, diabetes mellitus, liver cirrhosis, or cancer and those receiving prednisolone (PSL) or immunosuppressive therapy. The IBD group comprised individuals with a diagnosis of IBD based on clinical, endoscopic, radiologic, and histologic parameters.[9] Before endoscopy, all individuals are tested for HIV infection in our institution, and the HIV group comprised those study participants with a positive HIV test. Ultimately, a total of 89 individuals—26 in the IC group, 41 in the IBD group, and 22 in the HIV group, were selected for analysis.

The institutional review board at NCGM approved this study (approval number 2045). Written informed consent was obtained from all participants.

### Clinical factors and endoscopic findings

Clinical factors and endoscopic findings were reviewed from an electronic medical database (MegaOak, NEC, Tokyo, Japan). For the IBD group, we collected data on disease duration, extent of disease, disease activity, and medication on the day of colonoscopy. Disease activity was assessed by the Disease Activity Index (DAI)[10] in UC, and the International Organization for the study of Inflammatory Bowel Disease (IOIBD) endoscopic index[11] in CD. DAI score was divided into four categories of severity (0–2, 3–5, 6–10, and 10–12) as previously reported.[10] Treatment administered included 5-aminosalicylic, PSL, azathioprine, apheresis, tacrolimus, cyclosporine, infliximab, and adalimumab. PSL dose was the total amount received within 4 weeks prior to the day of endoscopy.[12]

### DNA extraction and PCR assay

DNA for PCR was extracted from ulcerative colonic mucosa obtained from participants at colonoscopy using a QIAamp DNA Mini Kit (Qiagen, Tokyo, Japan) according to the manufacturer’s instructions.

In some cases, the amount of extracted DNA was insufficient for quantitative real-time PCR for the eight HHVs. To evaluate the pathogenesis of gastrointestinal disease, we considered EBV, CMV, and HHV-8 more important than the other viruses, so we measured them by quantitative real-time PCR. We used the multiplex PCR method to detect HSV-1/2, HHV-6A/6B, and HHV-7 and conventional PCR to detect VZV [13]. Quantitative real-time PCR was performed using 50 ng DNA each and TaqMan Universal PCR Master Mix (Thermo Fisher Scientific, Kanagawa, Japan), with previously published sets of primers and probes for EBV and CMV,[14,15] and the 7900HT Fast Real Time PCR System (Thermo Fisher Scientific). Primers and probes for HHV-8 detection were: forward primer (5’- GAT TCC ACC ATT GTG CTC GAA T -3’, position: 47296–47317 of HHV-8 [U75698]), reverse primer (5’- TAC ACC AAC AGC TGC TGC AGA A -3’, position: 47396–47375), and probe (5’ [FAM]- ACG GAT TTG ACC TCG TGT TCC CCA TG -[TAMRA] 3’, position: 47321–47346). Thermal cycles were 50°C for 2 min and 95°C for 10 min, then 50 cycles of 95°C for 15 s, and 60°C for 60 s. Multiplex PCR was performed to amplify viral DNA of HSV-1/2, HHV-6A/6B, and HHV-7 using 50 ng of extracted DNA by the method reported by Tanaka et al.[13] with slight modification. Briefly, the KAPA2G Fast Multiplex PCR Kit (Kapa Biosystems, Inc., Wilmington, MA) was used with the following thermal cycles: 95°C for 3 min, then touchdown cycles of 95°C for 15 s, 70 to 61°C for 30 s (1°C decrease in temperature per cycle), and 72°C for 20 s, and finally 30 cycles of 95°C for 15 s, then 60°C for 30 s and 72°C for 20 s. Conventional PCR was performed separately for the detection of VZV-DNA using a published primer set[13] and 50 ng DNA with the following thermal cycles: 95°C for 10 min, then touchdown cycles of 95°C for 30 s, 70 to 61°C for 30 s (1°C decrease in temperature per cycle), and 72°C for 1 min, and finally 35 cycles of 95°C for 30 s, then 60°C for 30 s, and 72°C for 30 s. PCR products were evaluated by 3% NuSieve 3:1 Agarose electrophoresis (Lonza Japan, Tokyo, Japan). Detection limit of the copy number of each virus was 80 copies/μg DNA. Positive controls used for each viral DNA were purchased from Advanced Biotechnologies Inc. (Eldersburg, MD).

### Statistical analysis

We compared nominal variables or continuous variables between groups using the χ^2^, Fisher’s exact, Mann–Whitney *U*, or Kruskal–Wallis tests as appropriate. The χ^2^ test for trend was used to examine the HHV positivity rate in the order of the IC, IBD, and HIV groups. There were no IBD individuals with HSV-1, HSV-2, VZV, or HHV-8. Therefore, we performed multiple logistic regression analysis adjusting for age and sex among the three groups with EBV, CMV, HHV-6, or HHV-7. We also compared the HHV positivity rate between IBD individuals receiving immunosuppressive therapy and those not receiving it and between untreated HIV-infected individuals with low CD4 and treated HIV-infected individuals. Statistical significance was set at P < 0.05. All statistical analyses were performed using Stata software (version 14, Stata Co., College Station, TX).

## Results

### Baseline characteristics of IC, IBD, and HIV-infected individuals

**Table** 1. shows the characteristics of the three groups.

**Table 1:** Characteristics of the IC, IBD, and HIV-infected groups (N = 89).

Factors	IC group (n = 26)	IBD group (n = 41)	HIV-infected group (n = 22)
Age (range) years	50 (21–81)	42 (16–91)	40.5 (28–80)
Sex, male	15 (57.7%)	24 (58.5%)	22 (100%)
PSL	0	12 (29.3%)	1 (4.5%)
^a^Immunosuppressive therapy	0	7 (17.1%)	0
CD4 cell counts (cells/μL)			119.5 (4–1287)
AIDs status			14 (63.6%)
HIV VL (copies/mL)			^b^300,000 (110–5,000,000)
VL ≤ 50 (normal range)			6 (27.3%)
VL > 50			16 (72.7%)
Administration of HAART			10 (45.5%)
No HAART and CD4 < 100 (cells/μL)			8 (36.4%)

Note

^a^Immunosuppressive therapy is defined as prednisolone, azathioprine, or TNF-α inhibitor.

^b^300,000 (110–5,000,000) is the median (range) HIV viral load in 16 (72.7%) individuals with HIV VL > 50.

**Abbreviations**: IC, immunocompetent; IBD, inflammatory bowel disease; HAART, highly active antiretroviral therapy; HIV, human immunodeficiency virus; PSL, prednisolone; VL, viral load.

No significant difference in age was observed among the three groups (P = 0.17). No significant sex difference was observed between the IC and IBD groups (P = 0.20), and the HIV group comprised men only. None of the IC group had received PSL or immunosuppressive therapy. Twelve individuals in the IBD group (29.3%) received PSL and 7 (17.1%) received immunosuppressive therapy. One patient in the HIV group (4.5%) received PSL. In the HIV group, median CD4 cell count was 119.5/μl (range, 4-1287/μl) and 11 (50%) individuals had CD4 cell count < 100/μl. **Table 2** shows the characteristics of UC and CD individuals.

**Table 2:** Characteristics of the IBD group (n = 41).

	UC (n = 33)	CD (n = 8)	P
Disease duration			
< 1 year	15 (45.5%)	4 (50.0%)	0.38
1–5 years	7 (21.2%)	0	
> 5 years	10 (30.3%)	3 (37.5%)	
Unknown	1 (3.0%)	1 (12.5%)	
Extent of disease	Pancolitis 20 (60.6%)	Ileum 0	NA
	Left 13 (39.4%)	Colon 4 (50%)	
		Ileocolon 4 (50%)	
Disease activity			
DAI score in UC, mean ± SD	7.8 ± 3.1		
0–2	4 (12.1%)		
3–5	3 (9.1%)		
6–10	20 (60.6%)		
11–12	6 (18.2%)		
IOIBD score in CD, mean ± SD		2.5 ± 1.3	
1		2 (25.0%)	
2		3 (37.5%)	
3		0	
4		3 (37.5%)	
≥ 5		0	
Medication			
5-aminosalicylic acid	26 (78.8%)	5 (62.5%)	0.38
PSL	10 (30.3%)	2 (25.0%)	1.0
All PSL (mg) during 4W, median (IQR)	242.5 (140,880)	280 (240,320)	0.81
Azathioprine	5 (15.2%)	1(12.5%)	1.0
Apheresis	1 (3%)	0	1.0
Tacrolimus	0	0	NA
Cyclosporin	0	0	NA
Infliximab	1 (3%)	2 (25%)	0.092
Adalimumab	0	0	NA

**Abbreviations**: IBD, inflammatory bowel disease; UC, ulcerative colitis; CD, Crohn’s disease; DAI, Disease Activity Index; IOIBD, International Organization for the Study of Inflammatory Bowel Disease; IQR, interquartile range; PSL, prednisolone; SD, standard deviation; NA, not applicable.

No significant difference in disease duration or medication was found between UC and CD individuals. Twenty UC individuals (60.6%) had pancolitis and 4 CD individuals (50%) had ileocolic lesions. Mean DAI score was 7.8 in UC and 2.5 in CD.

### Mucosal HHV PCR positivity rate in IC, IBD, and HIV-infected individuals

**Fig 1:**
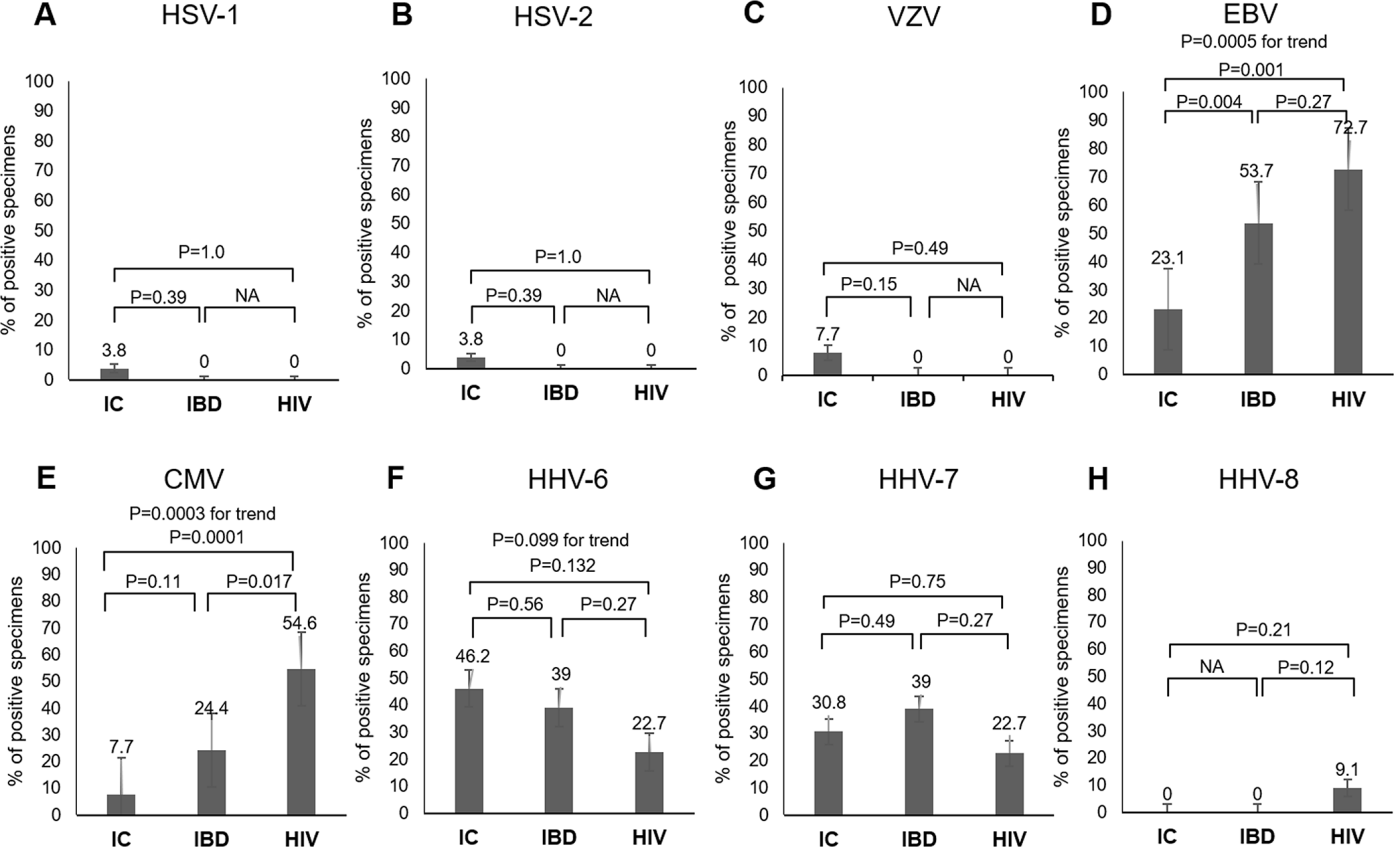
Mucosal HHV PCR positivity rate in the IC, IBD, and HIV-infected groups. Note: Bars represent standard error. Abbreviations: CMV, cytomegalovirus; EBV, Epstein–Barr virus; HHV, human herpesvirus; HIV, human immunodeficiency virus; HSV-1/2, herpes simplex virus-1/2; IBD, inflammatory bowel disease; IC, immunocompetent; NA, not applicable; VZV, varicella zoster virus.

**Fig 1** shows the positivity rate of the eight HHVs in colonic mucosa and a comparison between the three groups for HSV-1 (**Fig 1A**), HSV-2 (**Fig 1B**), VZV (**Fig 1C**), EBV **(Fig 1D**), CMV (**Fig 1E**), HHV-6 (**Fig 1F**), HHV-7 (**Fig 1G**), and HHV-8 (**Fig 1H**).

The HSV-1 positivity rate was 1.1% in all individuals and showed no significant difference between the three groups (3.8%, 0%, and 0% in the IC, IBD, and HIV groups, respectively). The HSV-2 positivity rate was 1.1% in all individuals and also showed no significant difference between the three groups (3.8%, 0%, and 0% in the IC, IBD, and HIV groups, respectively). Likewise, the VZV positivity rate was 2.2% in all individuals and showed no significant difference between the three groups (7.7%, 0%, and 0% in the IC, IBD, and HIV groups, respectively).

The EBV positivity rate was 49.4% in all individuals and showed a significantly increasing trend in the order of 23.1%, 53.7%, and 72.7% in the IC, IBD, and HIV groups, respectively (**Fig 1D**; P for trend = 0.0005). Multivariate analysis revealed a significantly increasing trend of EBV infection risk in the IBD group and HIV group compared with the IC group (Table 3; P for trend = 0.008).

**Table 3:** Crude and adjusted odds ratios of the infection risk of four HHVs in the IBD group and HIV group compared with the IC group.

Outcomes	Crude odds ratio (95% CI)	P	P for trend	Adjusted odds ratio (95% CI)	P	P for trend
EBV						
IC group	1 (referent)			1 (referent)		
IBD group	3.9 (1.3–11.6)	**0.016**		4.2 (1.3–13.2)	**0.014**	
HIV group	8.9 (2.4–33.0)	**0.001**	**0.004**	9.5 (2.2–40.9)	**0.003**	**0.008**
CMV						
IC group	1 (referent)			1 (referent)		
IBD group	3.9 (0.8–19.3)	0.099		5.2 (0.9–29.3)	0.059	
HIV group	14.4 (1.7–76.4)	**0.002**	**0.004**	20.8 (3.0–141.9)	**0.002**	**0.007**
HHV-6						
IC group	1 (referent)			1 (referent)		
IBD group	0.75 (0.28–2.0)	0.57		0.71 (0.25–2.0)	0.53	
HIV group	0.34 (0.097–1.2)	0.096	0.24	0.41 (0.97–1.7)	0.22	0.47
HHV-7						
IC group	1 (referent)			1 (referent)		
IBD group	1.4 (0.51–4.1)	0.49		1.2 (0.40–3.7)	0.73	
HIV group	0.66 (0.18–1.0)	0.53	0.42	0.57 (0.13–2.5)	0.46	0.51

**Note**: Bold indicates P < 0.05.

**Abbreviations**: CI, confidence interval; CMV, cytomegalovirus; EBV, Epstein–Barr virus; HHV, human herpesviruses; HIV, human immunodeficiency virus; IBD, inflammatory bowel disease; IC, immunocompetent.

The CMV positivity rate was 27.0% in all individuals and showed a significantly increasing trend in the order of 7.7%, 24.4%, and 54.5% in the IC, IBD, and HIV groups, respectively (**Fig 1E**; P for trend = 0.0003). Multivariate logistic analysis revealed a significantly increasing trend of CMV infection risk in the IBD group and HIV group compared with the IC group (**Table 3**; P for trend = 0.007).

The HHV-6 positivity rate was 37.1% in all individuals and showed a marginally significant decreasing tendency in the order of 46.2%, 39.0%, and 22.7% in the IC, IBD, and HIV groups, respectively (**Fig 1F**; P for trend = 0.099). Multivariate analysis revealed no significantly increasing trend of HHV-6 infection risk in the IBD group or HIV group compared with the IC group (**Table 3**; P for trend = 0.47).

The HHV-7 positivity rate was 32.6% in all individuals and 30.8%, 39.0%, and 22.7% in the IC, IBD, and HIV groups, respectively. Multivariate analysis revealed no significantly increasing trend of HHV-7 infection risk in the IBD group or HIV group compared with the IC group (**Table 3**; P for trend = 0.51).

The HHV-8 positivity rate was 2.2% in all individuals and 0%, 0%, and 3.8% in the IC, IBD, and HIV groups, respectively.

### Quantitative mucosal EBV PCR and CMV PCR in IC, IBD, and HIV-infected individuals

**Fig 2:**
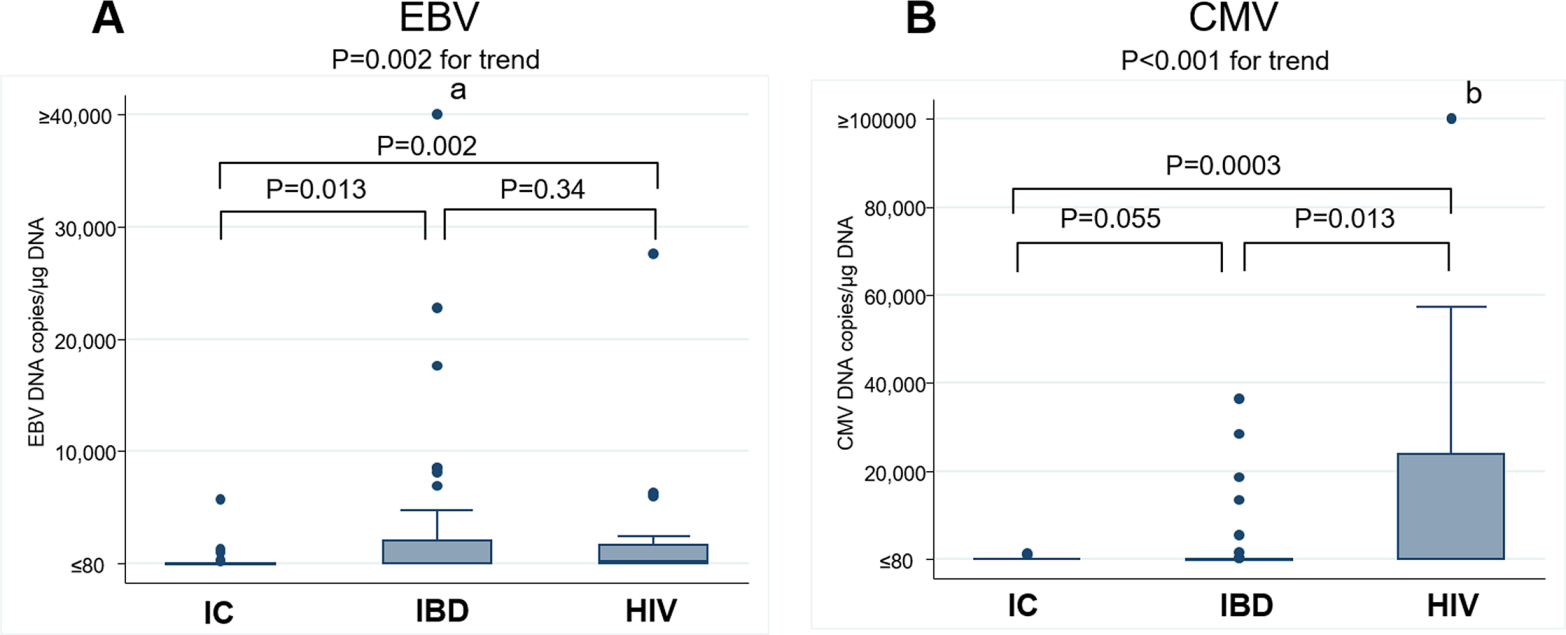
Quantitative mucosal EBV and CMV in the IC, IBD, and HIV-infected groups. **Note**: ^a^95,286 copies/μg DNA, ^b^172,000 and 498,000 copies/μg DNA. **Abbreviations**: CMV, cytomegalovirus; EBV, Epstein–Barr virus; HHV, human herpesvirus; HIV, human immunodeficiency virus; IBD, inflammatory bowel disease; IC, immunocompetent.

**Fig 2** shows the values of mucosal HHV PCR in colonic mucosa and the comparison between the three groups for EBV (Fig 2A) and CMV (Fig 2B). Both values showed a significantly increasing trend in the order of the IC, IBD, and HIV-infected groups.

Median mucosal EBV DNA values in the IC, IBD, and HIV groups were 0, 76, and 287 copies/μg DNA, respectively, showing a significant difference between the IC and IBD groups (P = 0.013) and between the IC and HIV groups (P = 0.002). Median mucosal CMV DNA values in the IC, IBD, and HIV groups were 0, 0, and 17 copies/μg DNA, respectively, for a significant difference between the IC and HIV groups (P = 0.0003) and between the IBD and HIV groups (P = 0.013). A significantly increasing trend was seen in the order of the IC, IBD, and HIV groups for values of mucosal EBV (P for trend = 0.002) and values of mucosal CMV (P for trend = 0.0001).

### Mucosal HHV PCR positivity rate in UC and CD individuals

**Fig 3:**
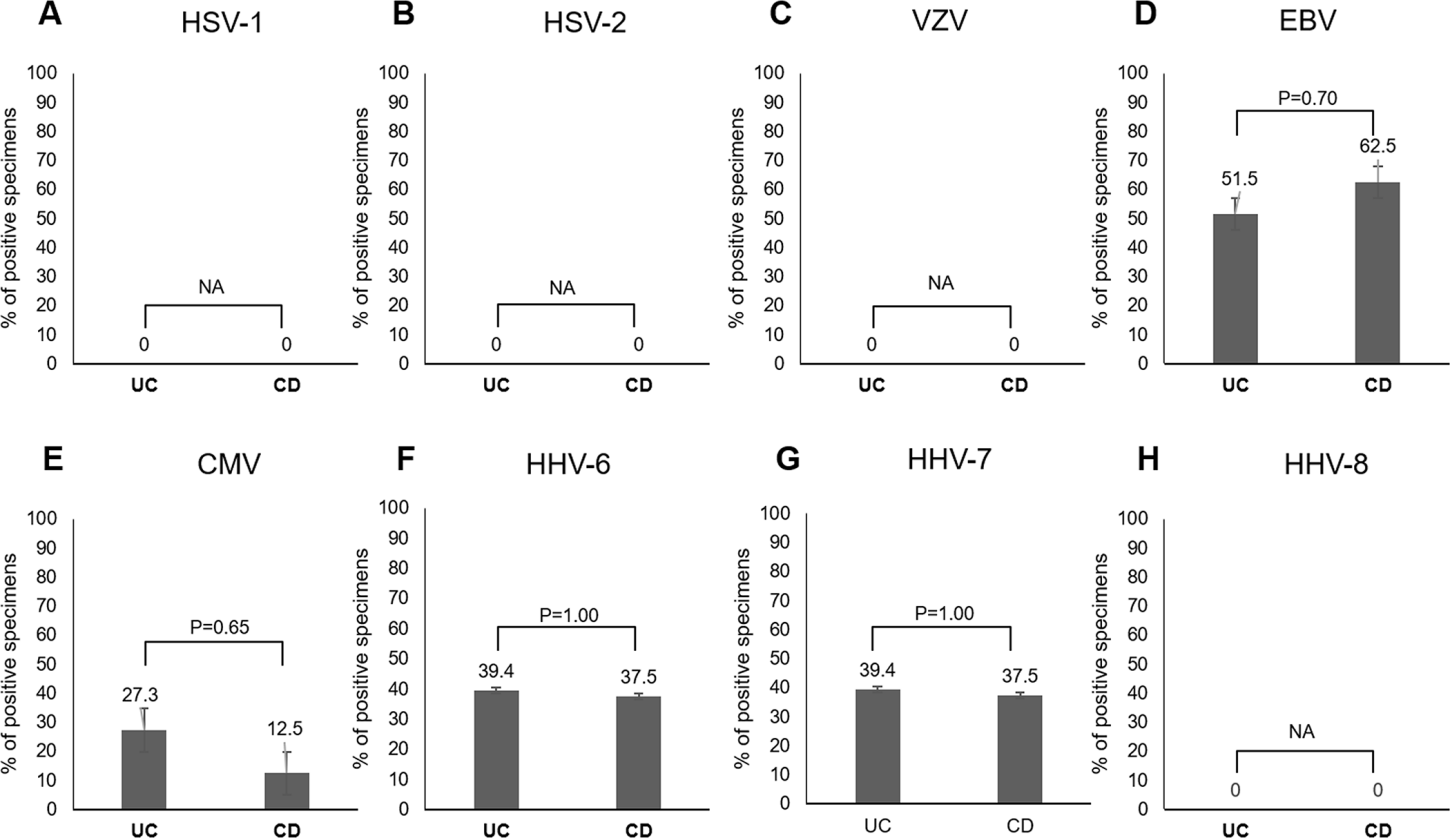
Mucosal HHV PCR positivity rate in UC and CD individuals (n = 41). **Note**: Bars represent standard error. **Abbreviations**: CMV, cytomegalovirus; EBV, Epstein–Barr virus; HHV, human herpesvirus; HIV, human immunodeficiency virus; HSV-1/2, herpes simplex virus-1/2; IBD, inflammatory bowel disease; IC, immunocompetent; NA, not applicable; VZV, varicella zoster virus.

**Fig 3** shows the positivity rate of the eight HHVs in colonic mucosa and a comparison between UC and CD individuals for HSV-1 (**Fig 3A**), HSV-2 (**Fig 3B**), VZV (**Fig 3C**), EBV (**Fig 3D**), CMV (**Fig 3E**), HHV-6 (**Fig 3F**), HHV-7 (**Fig 3G**), and HHV-8 (**Fig 3H**). No significant differences in the positivity rates of the eight HHVs were observed between the two groups.

### Mucosal HHV PCR positivity rate in IBD individuals with and without IS therapy

Of the 41 IBD individuals, 17 (41.5%) were included in the IS therapy group, defined as individuals taking PSL, AZA, or TNF-α inhibitors. **S1 Fig** shows the positivity rate of the eight HHVs in colonic mucosa and a comparison between the IS therapy group and the non-IS therapy group for HSV-1 (**S1A Fig**), HSV-2 (**S1B Fig**), VZV (**S1C Fig**), EBV (**S1D Fig**), CMV (**S1E Fig**), HHV-6 (**S1F Fig**), HHV-7 (**S1G Fig**), and HHV-8 (**S1H Fig**). The rate of positive EBV infection alone was significantly higher in the IS therapy group than in the non-IS therapy group (P = 0.025).

### Mucosal HHV PCR positivity rate in untreated HIV-infected individuals with low CD4 and treated HIV-infected individuals

There were 8 (36.4%) untreated HIV-infected individuals with low CD4 T (< 100 cells/μL). **S2 Fig** shows the positivity rate of the eight HHVs in colonic mucosa and a comparison between the untreated HIV-infected individuals with low CD4 T and the treated HIV-infected individuals for HSV-1 (**S2A Fig**), HSV-2 (**S2B Fig**), VZV (**S2C Fig**), EBV (**S2D Fig**), CMV (**S2E Fig**), HHV-6 (**S2F Fig**), HHV-7 (**S2G Fig**), and HHV-8 (**S2H Fig**). We found that the HHV-7 infection rate was significantly higher in the untreated HIV group with low CD4 than in the treated HIV group.

## Discussion

We used mucosal PCR assay to examine the detection rates of eight HHVs in colonic mucosa from colonoscopic biopsy specimens of ulcerated lesions in IC, IBD, and HIV-infected individuals. EBV, CMV, HHV-6, and HHV-7, but not HSV-1, HSV-2, VZV, and HHV-8, were identified in the colonic mucosa of IBD individuals. Also, the positivity rates of EBV and CMV, but not of the other HHVs, increased significantly in the order of the IC, IBD, and HIV groups. We confirmed this finding by quantitative PCR. There was no significant difference in positivity rate of the eight HHVs between UC and CD individuals.

The ECCO has reported various cases of HHV infection in IBD individuals, but no studies have comprehensively investigated the rates of mucosal infections in IBD.[8] Generally, after initial asymptomatic infection, many types of HHVs become latent in certain organs. HSV-1, HSV-2, and VZV reportedly remain in the ganglion, CMV in the glandular system, and EBV, HHV-6, HHV-7, and HHV-8 in the lymphatic tissues,[16,17] and they are reactivated as the host immune system weakens. Upon reactivation, HSV-1, HSV-2, and VZV are transported from the latently infected ganglia to the skin where the viruses will be shed via skin lesions.[16] This may explain why these viruses have not been detected in the colonic mucosa. Consistent with our findings, Wakefield et al. reported negative PCR results for HSV-1 and VZV in the colonic mucosa of all IBD individuals tested.[18] Conversely, in our study, colonic HHV-8 infection was confirmed in HIV-infected individuals alone. Lavagna et al. performed PCR to compare HHV infection status in colonic mucosa between IBD individuals and those who had undergone colonic cancer surgery (the control group) and found that HHV-8 was negative in all individuals in both groups.[19] This was also consistent with our findings, possibly due to the high risk of HHV-8 transmission through anal intercourse and saliva in men.[20] This study found CMV, EBV, HHV-6, and HHV-7 infection in the colonic mucosa of IBD individuals, probably due to latent infection of tissues (e.g. glandular and lymphatic tissues) in the intestinal tract.[16,17] CMV mainly infects monocytes, which differentiate into macrophages following stimulation by inflammatory cytokines.[17] During the differentiation and stimulation process, CMV can be reactivated and proceed to infect epithelial, vascular endothelial, and interstitial cells.[16] EBV mainly infects B-lymphocytes and can also be reactivated by various stimuli; reactivated EBV infects various cells including T lymphocytes, natural killer cells, and epithelial cells. It has been reported that HHV-6 infects monocytes and promyelocytes and that HHV-7 infects CD4+ T lymphocytes mainly; they tend to have a broader spectrum of infection following reactivation in response to stimuli. [16]

Previously, it was reported that positivity rates in IBD individuals for mucosal infection by EBV and CMV were 48.3–62.5%[21,22] and 10–36%,[23,24] respectively, which are consistent with our findings of 49.4% and 27.0%, respectively. Nahar et al. tested stool samples by PCR to compare positivity rates for HHV-1 to HHV-6 between an IC group and an immunocompromised group (including HIV infection) and found significantly higher positivity rates for both viruses in the latter group than in the former group (P < 0.05).[7] Based on these previous findings, it is highly likely that mucosal infection rates for EBV and CMV are higher in IBD and HIV-infected individuals than in IC individuals. There is no consensus on the differences in CMV infection rates between UC and CD individuals. McCurdy et al. performed histopathology of colonic mucosa and found that CMV infection rates were significantly higher in UC individuals (P = 0.003).[25] Nakase et al. showed differences in colonic immunity between the two groups; CD4+ T cells produce IFN-α that inhibits CMV reactivation in CD but not in UC individuals, resulting in suppressed reactivation of CMV in CD individuals.[26] Conversely, Ormeci et al. tested CMV infection by mucosal PCR but found no significant difference in infection rate between UC individuals (12%) and CD individuals (19%).[27] In the present study, CMV infection rates were slightly higher in the UC individuals, albeit no significance so, partly due to the limited number of cases tested. A future study including more cases is needed. In agreement with our study, a previous study showed no significant differences in colonic infection rates by HSV-1, VZV, EBV, and HHV-6 between UC and CD individuals.[18]

This study has several limitations. First, it is a single-center study. Second, all participants in the HIV group were men, so there was selection bias. Third, we had no information on the precise location or size of the ulcers and thus could not assess the association between ulcer location and HHV detection. Fourth, there was a low prevalence of HSV-1/2, VZV, and HHV-8 in the relatively small number of IBD individuals and IBD individuals showed heterogeneity in our study. Further studies with more IBD individuals are needed.

In conclusion, among the eight HHVs investigated, EBV, CMV, HHV-6, and HHV-7, but not HSV-1, HSV-2, VZV, or HHV-8, were identified in the colonic mucosa of IBD individuals. EBV and CMV in colonic mucosa was correlated with host immune status in increasing order of IC, IBD, and HIV-infected individuals.

## Supporting information

S1 Fig Mucosal HHV PCR positivity rate in the immunosuppressive (IS) and the non-IS therapy group (n = 41). **Note**: Bars represent standard error. **Abbreviations**: CMV, cytomegalovirus; EBV, Epstein–Barr virus; HHV, human herpesvirus; HIV, human immunodeficiency virus; HSV-1/2, herpes simplex virus-1/2; NA, not applicable; VZV, varicella zoster virus.
(TIF)

S2 Fig Mucosal HHV PCR positivity rate in the untreated HIV group with low CD4 and the treated HIV group (n = 22). **Note**: Bars represent standard error. **Abbreviations**: CMV, cytomegalovirus; EBV, Epstein–Barr virus; HHV, human herpesvirus; HIV, human immunodeficiency virus; HSV-1/2, herpes simplex virus-1/2; NA, not applicable; VZV, varicella zoster virus.
(TIF)

## References

[1] Weir JP. Genomic organization and evolution of the human herpesviruses. Virus Genes. 1998;16: 85–93. 9562893 10.1023/a:1007905910939

[2] Miller CS, Avdiushko SA, Kryscio RJ, Danaher RJ, Jacob RJ. Effect of prophylactic valacyclovir on the presence of human herpesvirus DNA in saliva of healthy individuals after dental treatment. J Clin Microbiol. 2005;43: 2173–2180. doi: 10.1128/JCM.43.5.2173-2180.2005 15872238 10.1128/JCM.43.5.2173-2180.2005 PMC1153765

[3] Sipponen T, Turunen U, Lautenschlager I, Nieminen U, Arola J, Halme L. Human herpesvirus 6 and cytomegalovirus in ileocolonic mucosa in inflammatory bowel disease. Scand J Gastroenterol. 2011;46: 1324–1333. doi: 10.3109/00365521.2011.605466 21879802 10.3109/00365521.2011.605466

[4] Xu XR, Liu CQ, Feng BS, Liu ZJ. Dysregulation of mucosal immune response in pathogenesis of inflammatory bowel disease. World J Gastroenterol. 2014;20: 3255–3264. doi: 10.3748/wjg.v20.i12.3255 24695798 10.3748/wjg.v20.i12.3255 PMC3964397

[5] Lawlor G, Moss AC. Cytomegalovirus in inflammatory bowel disease: pathogen or innocent bystander? Inflamm Bowel Dis. 2010;16: 1620–1627. doi: 10.1002/ibd.21275 20232408 10.1002/ibd.21275

[6] Ciccocioppo R, Racca F, Paolucci S, Campanini G, Pozzi L, Betti E, et al Human cytomegalovirus and Epstein-Barr virus infection in inflammatory bowel disease: need for mucosal viral load measurement. World J Gastroenterol. 2015;21: 1915–1926. doi: 10.3748/wjg.v21.i6.1915 25684960 10.3748/wjg.v21.i6.1915 PMC4323471

[7] Nahar S, Iraha A, Hokama A, Uehara A, Parrott G, Ohira T, et al Evaluation of a multiplex PCR assay for detection of cytomegalovirus in stool samples from patients with ulcerative colitis. World J Gastroenterol. 2015;21: 12667–12675. doi: 10.3748/wjg.v21.i44.12667 26640344 10.3748/wjg.v21.i44.12667 PMC4658622

[8] Rahier JF, Magro F, Abreu C, Armuzzi A, Ben-Horin S, Chowers Y, et al Second European evidence-based consensus on the prevention, diagnosis and management of opportunistic infections in inflammatory bowel disease. J Crohns Colitis. 2014;8: 443–468. doi: 10.1016/j.crohns.2013.12.013 24613021 10.1016/j.crohns.2013.12.013

[9] Nikolaus S, Schreiber S. Diagnostics of inflammatory bowel disease. Gastroenterology. 2007;133: 1670–1689. doi: 10.1053/j.gastro.2007.09.001 17983810 10.1053/j.gastro.2007.09.001

[10] Schroeder KW, Tremaine WJ, Ilstrup DM. Coated oral 5-aminosalicylic acid therapy for mildly to moderately active ulcerative colitis. A randomized study. N Engl J Med. 1987;317: 1625–1629. doi: 10.1056/NEJM198712243172603 3317057 10.1056/NEJM198712243172603

[11] Myren J, Bouchier IA, Watkinson G, Softley A, Clamp SE, de Dombal FT. The O.M.G.E. Multinational Inflammatory Bowel Disease Survey 1976–1982. A further report on 2,657 cases. Scand J Gastroenterol Suppl. 1984;95: 1–27. 6379849

[12] Matsuoka K, Iwao Y, Mori T, Sakuraba A, Yajima T, Hisamatsu T, et al Cytomegalovirus is frequently reactivated and disappears without antiviral agents in ulcerative colitis patients. Am J Gastroenterol. 2007;102: 331–337. doi: 10.1111/j.1572-0241.2006.00989.x 17156136 10.1111/j.1572-0241.2006.00989.x

[13] Tanaka T, Kogawa K, Sasa H, Nonoyama S, Furuya K, Sato K. Rapid and simultaneous detection of 6 types of human herpes virus (herpes simplex virus, varicella-zoster virus, Epstein-Barr virus, cytomegalovirus, human herpes virus 6A/B, and human herpes virus 7) by multiplex PCR assay. Biomed Res. 2009;30: 279–285. 19887724 10.2220/biomedres.30.279

[14] Beuselinck K, van Ranst M, van Eldere J. Automated extraction of viral-pathogen RNA and DNA for high-throughput quantitative real-time PCR. J Clin Microbiol. 2005;43: 5541–5546. doi: 10.1128/JCM.43.11.5541-5546.2005 16272483 10.1128/JCM.43.11.5541-5546.2005 PMC1287800

[15] Machida U, Kami M, Fukui T, Kazuyama Y, Kinoshita M, Tanaka Y, et al Real-time automated PCR for early diagnosis and monitoring of cytomegalovirus infection after bone marrow transplantation. J Clin Microbiol. 2000;38: 2536–2542. 10878039 10.1128/jcm.38.7.2536-2542.2000 PMC86962

[16] Gilden DH, Mahalingam R, Cohrs RJ, Tyler KL. Herpesvirus infections of the nervous system. Nat Clin Pract Neurol. 2007;3: 82–94. doi: 10.1038/ncpneuro0401 17279082 10.1038/ncpneuro0401

[17] Zhuravskaya T, Maciejewski JP, Netski DM, Bruening E, Mackintosh FR, St Jeor S. Spread of human cytomegalovirus (HCMV) after infection of human hematopoietic progenitor cells: model of HCMV latency. Blood. 1997;90: 2482–2491. 9310501

[18] Wakefield AJ, Fox JD, Sawyerr AM, Taylor JE, Sweenie CH, Smith M, et al Detection of herpesvirus DNA in the large intestine of patients with ulcerative colitis and Crohn's disease using the nested polymerase chain reaction. J Med Virol. 1992;38: 183–190. 1287131 10.1002/jmv.1890380306

[19] Lavagna A, Bergallo M, Daperno M, Sostegni R, Ravarino N, Crocella L, et al The hazardous burden of Herpesviridae in inflammatory bowel disease: the case of refractory severe ulcerative colitis. Dig Liver Dis. 2006;38: 887–893. doi: 10.1016/j.dld.2006.07.011 16931197 10.1016/j.dld.2006.07.011

[20] Antman K, Chang Y. Kaposi's sarcoma. N Engl J Med. 2000;342: 1027–1038. doi: 10.1056/NEJM200004063421407 10749966 10.1056/NEJM200004063421407

[21] Nissen LH, Nagtegaal ID, de Jong DJ, Kievit W, Derikx LA, Groenen PJ, et al Epstein-Barr virus in inflammatory bowel disease: the spectrum of intestinal lymphoproliferative disorders. J Crohns Colitis. 2015;9: 398–403. doi: 10.1093/ecco-jcc/jjv040 25740811 10.1093/ecco-jcc/jjv040

[22] Yanai H, Shimizu N, Nagasaki S, Mitani N, Okita K. Epstein-Barr virus infection of the colon with inflammatory bowel disease. Am J Gastroenterol. 1999;94: 1582–1586. doi: 10.1111/j.1572-0241.1999.01148.x 10364028 10.1111/j.1572-0241.1999.01148.x

[23] Leveque N, Brixi-Benmansour H, Reig T, Renois F, Talmud D, Brodard V, et al Low frequency of cytomegalovirus infection during exacerbations of inflammatory bowel diseases. J Med Virol. 2010;82: 1694–1700. doi: 10.1002/jmv.21877 20827767 10.1002/jmv.21877

[24] Cottone M, Pietrosi G, Martorana G, Casa A, Pecoraro G, Oliva L, et al Prevalence of cytomegalovirus infection in severe refractory ulcerative and Crohn's colitis. Am J Gastroenterol. 2001;96: 773–775. doi: 10.1111/j.1572-0241.2001.03620.x 11280549 10.1111/j.1572-0241.2001.03620.x

[25] McCurdy JD, Jones A, Enders FT, Killian JM, Loftus EV Jr, Smyrk TC, et al A model for identifying cytomegalovirus in patients with inflammatory bowel disease. Clin Gastroenterol Hepatol. 2015;13: 131–7; quiz e7. doi: 10.1016/j.cgh.2014.05.026 24993369 10.1016/j.cgh.2014.05.026

[26] Nakase H, Yoshino T, Honzawa Y, Chiba T. Low prevalence of CMV infection in patients with Crohn's disease in comparison with ulcerative colitis: effect of different immune response on prevalence of CMV infection. Dig Dis Sci. 2010;55: 1498–1499. doi: 10.1007/s10620-010-1162-0 20198427 10.1007/s10620-010-1162-0

[27] Ormeci AC, Akyuz F, Baran B, Soyer OM, Gokturk S, Onel M, et al Steroid-refractory inflammatory bowel disease is a risk factor for CMV infection. Eur Rev Med Pharmacol Sci. 2016;20: 858–865. 27010142

