# Supplementary figures and images for: PCR detection of human herpesviruses in colonic mucosa of individuals with inflammatory bowel disease: Comparison with individuals with immunocompetency and HIV infection
Source: PLoS One. 2017 Sep 13;12(9):e0184699. doi: 10.1371/journal.pone.0184699 (PMC5597220; doi:10.1371/journal.pone.0184699)

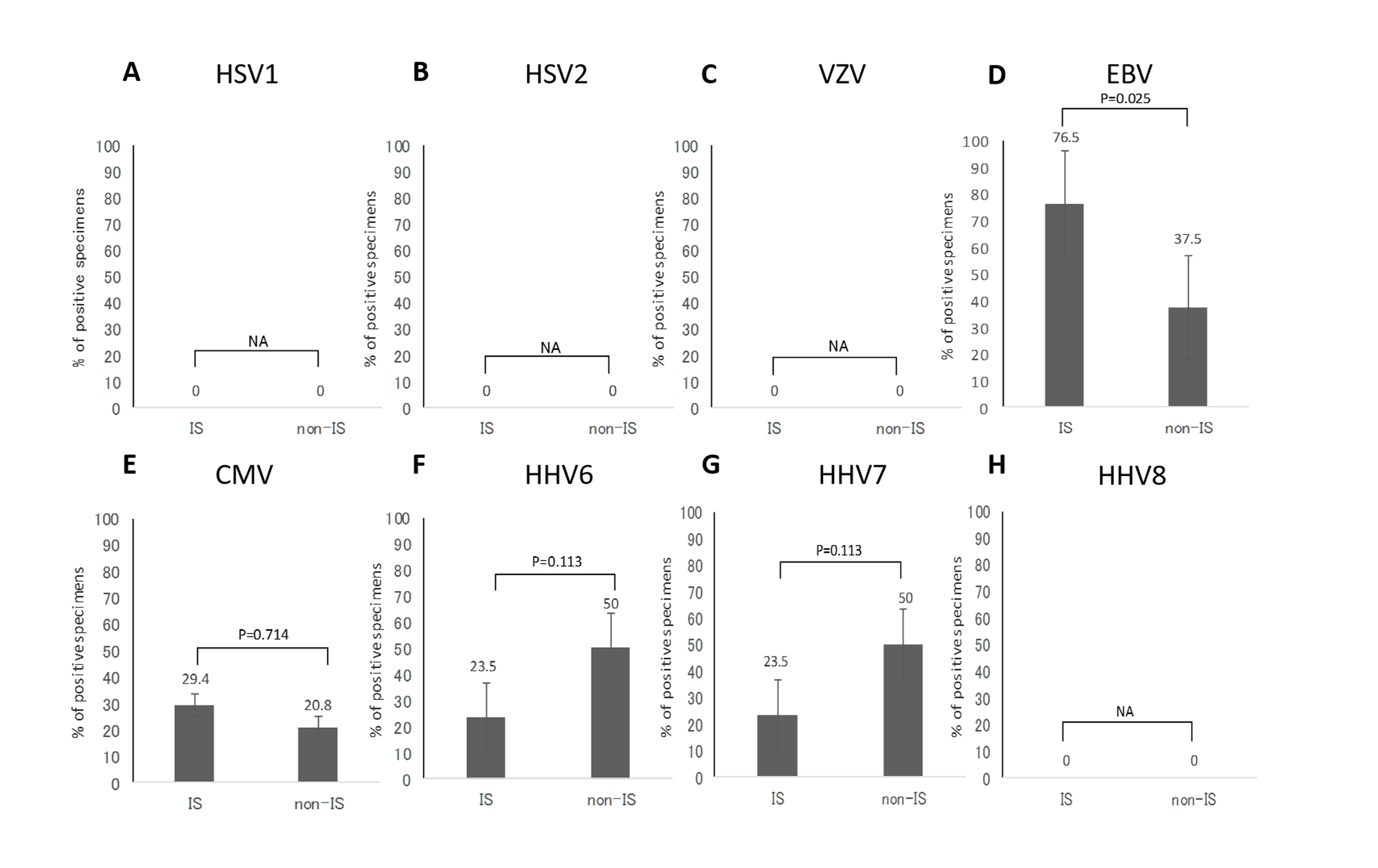

Supplement: S1 Fig — Note: Bars represent standard error. Abbreviations: CMV, cytomegalovirus; EBV, Epstein–Barr virus; HHV, human herpesvirus; HIV, human immunodeficiency virus; HSV-1/2, herpes simplex virus-1/2; NA, not applicable; VZV, varicella zoster virus. (TIF) [file pone.0184699.s001.tif]

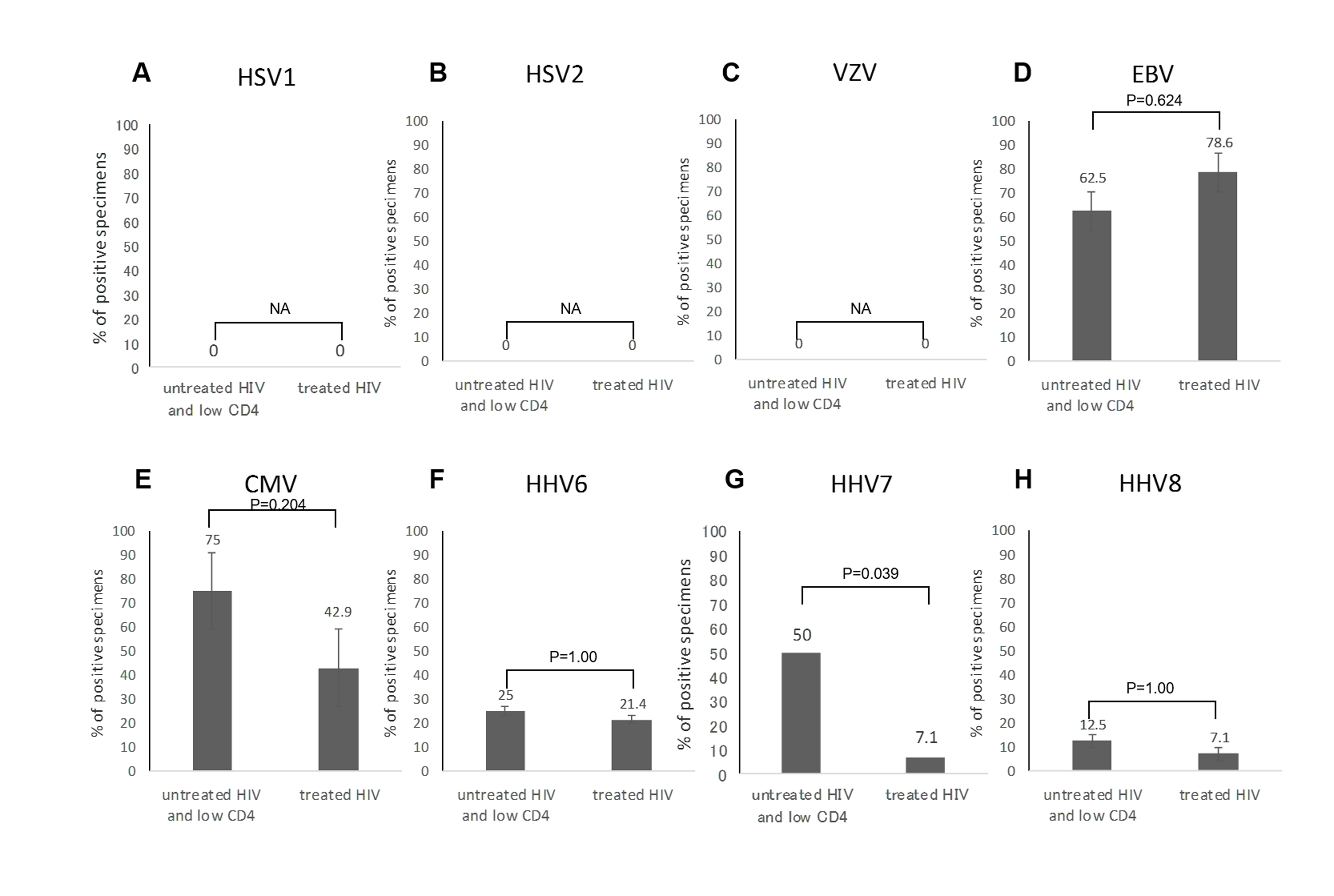

Supplement: S2 Fig — Note: Bars represent standard error. Abbreviations: CMV, cytomegalovirus; EBV, Epstein–Barr virus; HHV, human herpesvirus; HIV, human immunodeficiency virus; HSV-1/2, herpes simplex virus-1/2; NA, not applicable; VZV, varicella zoster virus. (TIF) [file pone.0184699.s002.tif]
